# How Important Are Social Support, Expectations and Coping Patterns during Cardiac Rehabilitation

**DOI:** 10.1155/2014/973549

**Published:** 2014-09-15

**Authors:** Maria J. C. Blikman, Hege R. Jacobsen, Geir Egil Eide, Eivind Meland

**Affiliations:** ^1^Department of Global Public Health and Primary Care, Research Group of General Practice, University of Bergen, Kalfarveien 31, 5018 Bergen, Norway; ^2^Centre for Clinical Research, Haukeland University Hospital, Armauer Hansen's House, Bergen, Norway; ^3^Department of Global Public Health and Primary Care, Research Group of Lifestyle Epidemiology, University of Bergen, Bergen, Norway

## Abstract

*Purpose.* To investigate the predictive role of relevant social and psychosocial determinants on emotional distress among patients after cardiac rehabilitation. *Methods.* A longitudinal prospective study examined short-term (6 months) and long-term (2 years) impact of predictors on anxiety and depression complaints in 183 patients with 6-months follow-up data attending a four-week rehabilitation stay at the Krokeide Centre in Bergen, Norway. The patients mainly suffered from coronary heart disease. Emotional distress, coping, social support, socioeconomic status, and negative expectations were measured by means of internationally validated questionnaires. A composite score of anxiety and depression complaints was used as the outcome measure in the study. *Results.* This study revealed that task-oriented coping improved emotional status in long-term followup, and negative expectations were associated with emotional distress in short-term followup. A higher socioeconomic status and more social support predicted improved emotional status in short- as well as long-term followup. *Conclusions.* Fewer negative expectations and functional coping along with social support are important factors for the prevention of emotional distress after cardiac disease. Such elements should be addressed and encouraged in patients during cardiac rehabilitation.

## 1. Introduction

Coronary heart disease (CHD) is a common disease in the western world, including Norway [1]. CHD can be regarded as a traumatic event with concomitant emotional distress for the patients afflicted [2], and patients commonly develop psychological problems after experiencing CHD, including depression [3, 4]. A large amount of evidence shows a clear association between psychological health (including anxiety and depression) and the pathogenesis of cardiovascular disease in the sense that poor psychological health is a risk factor for developing CHD including recurrent CHD [5–9]. A broader understanding of factors influencing a healthy adaptation and coping is needed, not only to improve the quality of life, but also to limit future risk for CHD. The clinicians engaged in cardiac rehabilitation play a pivotal role in promoting a healthy adaptation.

Coping is assessable by means of self-report inventories and measures [10]. Lazarus and Folkman [11] originally distinguished two major functions of coping: problem-focused and emotion-focused. Later work suggested that three broad coping strategies are important: task, emotion, and avoidance strategies, which formed the basis for the more well-known General Coping Questionnaire [12]. Studies that pertain to this dimension during cardiac rehabilitation (CR) are sparse. Although the research literature is mostly focused on domain specific self-efficacy beliefs, general efficacy beliefs and expectations seem relevant, also among cardiac patients [13].

We wanted to identify other social and psychosocial factors influencing healthy adaptation and thereby psychological health after a coronary event. Low socioeconomic status (SES) is related to poor health including a higher risk of CHD [14]. This association can be explained by more environmental challenges and fewer psychosocial resources, which leads to the expectation of negative outcomes, loss of coping ability, strain, hopelessness, and chronic stress [14, 15]. In addition, a higher amount of experienced social support leads to less anxiety and depression in a group of patients who underwent coronary bypass grafting [16]. Furthermore, negative expectations are associated with depression in a group of heart failure patients [17]. We therefore hypothesized that expectancy beliefs, experienced social support, SES, and coping patterns were important factors influencing psychological health for patients after cardiac disease.

The objective of the present study was, accordingly, to examine the impact of social and psychosocial factors on emotional status at short- and long-term followup.

## 2. Patients and Methods

Two hundred and sixty-six patients attending a four-week CR programme at Krokeide Rehabilitation Centre (outside Bergen, Norway) were invited to participate in a clinical controlled trial during the years 2000 to 2002. Followup data were recorded until 2004. Recruitment, enrolment, and dropouts are described in a former study [18]. Sixty-one per cent had suffered a myocardial infarction (MI). Most of the others suffered from angina pectoris. We obtained followup data for 183 and 176 patients who were eligible for six months and two-year followup analyses, respectively. The baseline data of our patient group are presented in Table 1. In this observational study we combined the cohorts of the randomized controlled trial (RCT) and did not compare the two groups. The intervention in the RCT aimed primarily at lifestyle improvements and did not lead to significant group differences concerning emotional status (*t*-test *P*: 0.4 at both short- and long-term followup). Dropout rates were highest among young participants and people with high level of emotional distress [18].

An Anxiety-Depression-Irritability questionnaire measured emotional status/distress. The questionnaire is a 12-item semantic differential scale, exploring the present emotional status with pairs of words like “frightened”—“courageous” (anxiety) and “unhappy”—“happy” (depression), rated with a seven-point Likert scale. In studies of Norwegian CHD patients the questionnaire has shown good reliability and validity [4]. The anxiety and depression dimensions were strongly correlated (Pearson correlation: 0.77) and were therefore combined as a measure of emotional status. Due to low reliability the irritability dimension was left out, leaving an eight-item combined Cronbach's alpha of 0.88 for the anxiety and depressive complaints (Table 1). The combined score was used as the emotional status dependent variable. Increasing values indicated increasing emotional distress.

The questionnaires used for the independent variables in our study were composed of various internationally used and validated measures. Coping styles, health expectations, amount of received social support, and a variety of demographic factors were measured at inclusion. We evaluated the corresponding reliabilities by means of Cronbach's alphas (Table 1). The different coping styles were evaluated by means of the General Coping Questionnaire (GCQ-30), measuring task-focused, emotion-focused, and avoidance-focused coping strategies, which was developed on a conceptual basis by Joseph et al. [12]. This questionnaire rates each item on a four-point Likert scale from “never” to “very often.”

Self-reported household income was chosen as the SES predictor. Five different income categories were presented for the participants. Negative expectations were measured by means of a General Expectancy (GE) Questionnaire, adapted from the seven-item Positive Expectation Subscale [13]. The descriptive data, the reliability, and the construct validity of the current GE measure have previously been presented and established [18]. The amount of experienced social support was measured by five questions pertaining to the experience of being understood, closeness in relations with family members, closeness with friends, emotional acceptance in relations, and support from others. The items pertain to the functional aspects of social support and are adapted from a questionnaire used in research among breast cancer and heart transplant patients [19]. Each question was rated on a seven-point Likert scale and the overall mean was computed for each participant. The reliability of the measure is satisfactory (Table 1).

### 2.1. Statistical Analyses

We used Cronbach's alpha to measure the internal consistency and thereby the reliability of our survey instruments (Table 1). In our study the GCQ-30 showed suboptimal Cronbach's alphas for the task (0.69), emotion (0.60), and avoidance (0.62) questions. These are the values obtained after optimization by means of leaving out one and two questions that showed low internal consistency, for these two constructs. The suboptimal reliability of the Norwegian translation of the GCQ-30 questionnaire has also been demonstrated in a former study [20].

We explored to what extent coping patterns, SES, and other social and psychosocial factors influenced psychological health at short- (six months) and long-term (24 months) followup. For this, multiple linear regression analysis was used. Firstly, regression of emotional status on each predictor at six and 24 months, respectively, was done adjusting for gender and age by including these in the models. Secondly, all the predictors, including gender and age, were included in the multiple regression model, and a backward stepwise selection of predictors was performed excluding the least significant predictor with *P* > 0.05 at each step until a final model with only significant predictors was obtained. Gender and age were forced into all models. We performed the full model analyses in order to control the interrelations between the independent variables. The results were reported with estimated regression coefficients (*B*), 95% confidence intervals (CI), and *P* values. Preconditions for linear regression analyses were satisfactory. Analyses of residuals from the regression analyses showed no serious deviations from normality. The intercorrelations between the independent variables showed absolute values <0.51 (Pearson's correlation −0.50 between social support and negative expectations). Accordingly, multicollinearity can hardly invalidate the results from the multivariable analyses. *P* values ≤ 0.05 were considered as statistically significant. SPSS (version 18.0) was used for all statistical analyses with the GLM procedure for linear regression.

### 2.2. Ethics

The Regional Committee for Medical Research Ethics, Health Region III, and the Norwegian Data Inspectorate approved the study.

## 3. Results

In total, 80% of the participants were men. The mean age was 55 years for both genders. The average household income was about 400.000 NOK (70.000 USD) yearly.

Table 2 reports the short-term (six months) prognostic influence of both psychological and social factors on emotional status. The first columns demonstrate the impact of each predictor variable adjusted for gender and age. Regarding the psychological predictors, a task-oriented coping style was related to a lower level of emotional distress (*B* = −0.58, *P* = 0.010). An avoidant coping style predicted a higher degree of emotional distress (*B* = 0.57, *P* = 0.01). An emotional-oriented coping style showed no significant association with emotional distress. Negative expectations were related to impaired emotional status: *B* = 0.50, *P* < 0.001. In the final, full model, negative expectations (*B* = 0.25, *P* = 0.01) maintained significant prognostic associations with emotional distress.

Table 2 also shows that among the social determinants a higher degree of experienced social support predicted an improved emotional status at short-term followup (*B* = −0.59, *P* < 0.001). A higher household income was related to improved emotional status (*B* = −0.34, *P* < 0.001). Marital or cohabiting status predicted less emotional distress (*B* = −0.82, *P* = 0.001). In the final, full model, social support and household income remained significantly associated with emotional distress (*B* = −0.53, *P* < 0.001; *B* = −0.21, *P* = 0.001, resp.). Male gender was associated with impaired emotional status in short-term followup (*B* = 0.41, *P* = 0.04),

Table 3 demonstrates that a task-oriented coping style predicts less emotional distress at 24 months of followup (*B* = −0.97, *P* < 0.001). An avoidant coping style, although significantly related to short-term prognosis of emotional status, was only borderline significant at long-term followup (*B* = 0.38, *P* < 0.10). An emotional-oriented coping style showed no significant association with emotional distress either at short- or long-term followup. Negative expectations were also at long-term followup strongly associated with emotional distress (*B* = 0.36, *P* < 0.001) but in the full model analysis only proved borderline significant (*P* = 0.07). However, a task-oriented coping style remained significantly associated with improved emotional status at long-term followup (*B* = −0.79, *P* < 0.001) in the model with all independent variables entered.

Table 3 also reports that among the social factors, social support and household income are strongly related with improved emotional status at long-term followup (*B* = −0.46, *P* < 0.001 and *B* = −0.35, *P* < 0.001, resp.). Marital and cohabiting status predicted less emotional distress at 24 months of followup (*B* = −0.59, *P* = 0.01). We also observe from Table 3 that in the final, full model, social support and household income remain significantly associated with emotional distress at long-term followup (*B* = −0.33, *P* = 0.001; *B* = −0.23, *P* < 0.001). Emotional status improved with increasing age (*B* = −0.029, *P* = 0.001) at two years of followup.

## 4. Discussion

This study revealed that task-oriented coping improved emotional status in long-term followup, and negative expectations predicted emotional distress in short-term followup when we controlled the other variables. Increasing SES and social support improved emotional status in short- and long-term followup. The study confirmed that a lower household income and lack of social support are independent prognostic factors also when we controlled differences in coping styles, expectations, and other factors. Only a slight attenuating effect was observed in the multiple regression analyses with all variables entered in the model.

### 4.1. Psychological Predictors

Previous research has indicated that avoidant coping strategies lead to more emotional distress and that a task-focused coping style is negatively related or unrelated to anxiety and depression [17, 21, 22]. However, these studies also found that an emotional coping style associates with more psychological problems. We were unable to confirm this. Although our measure of emotional coping showed unsatisfactory reliability with concomitant danger of type II error, we observed regression coefficients close to zero. Unlike the present study, Endler and coworkers found that an avoidant coping style was not related to more psychological distress [10]. These inconsistencies may be explained by different characteristics of the patient groups included in the respective studies, the use of diverse coping scales, the fact that most coping scales have psychometric limitations, and differences in followup time [10, 17].

In former studies from this project specific expectancy beliefs (self-efficacy) concerning future lifestyle changes predicted health behavior changes more reliably than general expectancy. The influence of general expectancy also in these studies decreased significantly with time [23, 24]. It seems from the current results that general expectancy beliefs are important, but first and foremost in short-term followup.

In spite of some inconsistencies in the research literature, our study confirms that task-focused coping improves emotional status, whereas avoidant coping hampers psychological adaptation at least in short-term followup. Likewise, negative expectations strongly hamper emotional adaptation in CR patients. This is inline with a previous study including a population of heart failure patients [17]. Furthermore, negative expectations have been associated with long-term mortality in CHD patients [25].

### 4.2. Social Predictors

The association between low SES and poor health, including CHD, is well established [14, 26–29]. Psychosocial factors are suggested as important mediators for these effects [15]. However, such factors do not account for all of the SES impact on cardiovascular health [27]. Our study confirms that psychosocial factors, although important for emotional adaptation, do not account for the impact of SES on emotional health. In the present study coping, expectations, and social support only had a slightly attenuating effect on the relations between SES and emotional adaptation during followup.

The amount and quality of experienced social support also contributed to emotional health, as shown by our finding that social support predicts less emotional distress at both short- and long-term followup. This concurs with earlier studies among patients after coronary artery bypass grafting [16, 30]. However, a recent review article reported more conflicting results concerning the relationship between social support and emotional distress [31]. The absence of social or marital support is nevertheless a significant risk factor for poor prognoses in cardiac patients [32].

Some researchers claim that material and income differences are the main explanation for health inequality among people with different SES [33]. An increasing number of studies indicate, however, that psychosocial factors such as lack of control, anxiety, insecurity, depression, and lack of social affiliations are more relevant than the actual income difference [34]. General expectancy, social support, and adequate coping are important factors also in the present study, but SES seems to impact the emotional health significantly even when we adjust for these factors.

### 4.3. Clinical Relevance

Although the ENRICHD trial intervention did not improve the cardiovascular prognosis for depressed patients with CHD, cognitive behaviour therapy significantly improved emotional status [35] and quality of life [36] as compared with standard treatment. These effects are important rehabilitation measures that challenge clinicians also to attend to patients' cognitive interpretations in order to achieve beneficial psychosocial functioning. Emotional distress limits vitality and impairs the ability to return to work after cardiac disease [37]. Recent development in cognitive treatment methodology emphasizes functional coping in order to help patients develop alternatives to avoidant behaviour and strengthen their commitment to important values and goals in life [38]. The findings of the present study may support that this is relevant also in CR.

### 4.4. Limitations and Strengths of This Study

The low reliability particularly of emotional and avoidant coping styles introduces measurement “noise” for these constructs, especially for emotional coping style. The study is based on self-reported data and is prone to self-report bias. The strength of our study is that it concerns an important and prevalent clinical problem where knowledge is sparse concerning coping and its prognostic impact on health during CR. Explained variances are high and demonstrate that we have included relevant measures.

## 5. Conclusion

Fewer negative expectations and functional coping along with social support are associated with improved emotional health after cardiac disease. Such elements should be addressed and encouraged in patients during cardiac rehabilitation.

## Figures and Tables

**Table 1:** Baseline data on 183 patients with valid six-month data participating in a four-week cardiac rehabilitation programme at Krokeide Rehabilitation Centre, Bergen, Norway, from 2000 to 2002.

Variables	Total	Missing	Cronbach's *α*
Males, % (*n*)	80.3 (146)	0 (0)	
Age in years, mean (SD)	55.1 (9.2)	0 (0)	
Married/cohabiting			
Yes, % (*n*)	83.8 (151)	1.6 (3)	
No, % (*n*)	16.2 (29)		
Household income, mean (SD)	3.5^a^ (1.3)	14.2 (26)^b^	
Social support^c^, mean (SD)	5.4 (0.9)	0 (0)	0.74
Emotional status^d^, mean (SD)	3.0 (1.2)	0 (0)	0.88
Coping^e^			
Task, mean (SD)	2.5 (0.4)	0 (0)	0.69
Emotion, mean (SD)	2.2 (0.3)	0 (0)	0.60
Avoid, mean (SD)	2.0 (0.4)	0 (0)	0.62
Negative expectations^d^, mean (SD)	2.2 (1.0)	0 (0)	0.73

*n*: subsample size; SD: standard deviation.

^a^3.5 ≈ 400,000 NOK (70.000 USD) (scales 1–5: 3 = 301,000–400,000 NOK, 4 = 401,000–500,000 NOK).

^b^Household income data were obtained after conclusion of the study (at 24 months) and were completed for all participants with valid followup data.

^c^Total mean score social support (scales 1–7).

^d^Total mean score of anxiety and depression complaints (scales 1–7).

^e^Total mean score coping style (scales 1–4).

**Table 2:** Multiple linear regression of coping styles and other predictive factors on emotional status (anxiety and depression complaints) measured at short-term followup (6 months) at Krokeide Rehabilitation Centre, Bergen, Norway, included from 2000 to 2002.

Variables	*n*	Adjusted models^a^			Final model (*n* = 182)^b^		
		*B*	95% CI	*P* value	*B*	95% CI	*P* value^c^
Age (in years)							0.26
Gender (male/female)					0.41	(0.01, 0.82)	0.04
Employed (yes/no)	161	0.10	(−0.36, 0.55)	0.68			0.84
Cohabiting (yes/no)	180	−0.82	(−1.28, −0.36)	0.001			0.31
Household income	157	−0.34	(−0.49, −0.20)	<0.001	−0.21	(−0.33, −0.08)	0.001
Coping style							
Task	183	−0.58	(−1.01, −0.14)	0.01			0.16
Emotion	183	0.01	(−0.41, 0.44)	0.96			0.75
Avoid	183	0.57	(0.14, 1.01)	0.01			0.91
Social support	183	−0.59	(−0.76, −0.43)	<0.001	−0.53	(−0.72, −0.33)	<0.001
Negative expectations	183	0.50	(0.33, 0.66)	<0.001	0.25	(0.1, 0.4)	0.01
Intercept					5.13	(3.7, 6.6)	<0.001
Explained variance^d^ (*R* ^2^)							
Nonadjusted						0.36	
Adjusted						0.34	

*n*: subsample size; *B*: estimated regression coefficient; CI: confidence interval.

^a^Eight models adjusted for age and gender.

^b^Obtained by backward stepwise selection.

^c^The nonsignificant *P* values were retrieved from the excluded variables table of the backward stepwise selection analysis.

^d^ *R* ^2^ for final model.

**Table 3:** Multiple linear regression of coping styles and other predictive factors on emotional status (anxiety and depression complaints) measured at long-term followup (24 months) at Krokeide Rehabilitation Centre, Bergen, Norway, included from 2002–2004.

Variables	*n*	Adjusted models^a^			Final model (*n* = 173)^b^		
		*B*	95% CI	*P* value	*B*	95% CI	*P* value^c^
Age (in years)					−0.03	(0.05, 0.01)	0.001
Gender (male/female)					0.32	(−0.06, 0.70)	0.10
Employed (yes/no)	173	−0.02	(−0.45, 0.41)	0.92			0.17
Cohabiting (yes/no)	170	−0.59	(−1.05, −0.13)	0.01			0.51
Household income	168	−0.35	(−0.48, −0.21)	<0.001	−0.23	(−0.36, −0.11)	<0.001
Coping style							
Task	174	−0.97	(−1.40, −0.54)	<0.001	−0.79	(−1.22, −0.36)	<0.001
Emotion	174	−0.16	(−0.61, 0.28)	0.47			0.23
Avoid	174	0.38	(−0.07, 0.83)	<0.10			0.18
Social support	174	−0.46	(−0.64, −0.29)	<0.001	−0.33	(−0.52, −0.15)	0.001
Negative expectations	174	0.36	(0.19, 0.53)	<0.001	0.17	(−0.01, 0.36)	0.07
Intercept					7.95	(6.02, 9.87)	<0.001
Explained variance^d^ (*R* ^2^)							
Nonadjusted						0.36	
Adjusted						0.34	

*n*: subsample size; *B*: estimated regression coefficient; CI: confidence interval.

^a^Eight models adjusted for age and gender.

^b^Obtained by backward stepwise selection.

^c^The nonsignificant *P* values were retrieved from the excluded variables table of the backward stepwise selection analysis.

^d^ *R* ^2^ for final model.
